# DIP/WISH deficiency enhances synaptic function and performance in the Barnes maze

**DOI:** 10.1186/1756-6606-4-39

**Published:** 2011-10-21

**Authors:** Suhail Asrar, Keiko Kaneko, Keizo Takao, Jaina Negandhi, Makoto Matsui, Koji Shibasaki, Tsuyoshi Miyakawa, Robert V Harrison, Zhengping Jia, Michael W Salter, Makoto Tominaga, Tomoko Fukumi-Tominaga

**Affiliations:** 1 Neuroscience & Mental Health Program, The Hospital for Sick Children, Toronto, Canada; 2 Division of Cell Signaling, Okazaki Institute for Integrative Bioscience (National Institute for Physiological Sciences), Okazaki, Japan; 3 Center for Genetic Analysis of Behavior, National Institute for Physiological Sciences, Okazaki, Japan; 4 Department of Physiological Sciences, The Graduate University for Advanced Studies, Okazaki, Japan

## Abstract

**Background:**

DIP (diaphanous interacting protein)/WISH (WASP interacting SH3 protein) is a protein involved in cytoskeletal signaling which regulates actin cytoskeleton dynamics and/or microtubules mainly through the activity of Rho-related proteins. Although it is well established that: 1) spine-head volumes change dynamically and reflect the strength of the synapse accompanying long-term functional plasticity of glutamatergic synaptic transmission and 2) actin organization is critically involved in spine formation, the involvement of DIP/WISH in these processes is unknown.

**Results:**

We found that DIP/WISH-deficient hippocampal CA1 neurons exhibit enhanced long-term potentiation via modulation of both pre- and post-synaptic events. Consistent with these electrophysiological findings, DIP/WISH-deficient mice, particularly at a relatively young age, found the escape hole more rapidly in the Barnes maze test.

**Conclusions:**

We conclude that DIP/WISH deletion improves performance in the Barnes maze test in mice probably through increased hippocampal long-term potentiation.

## Background

DIP (diaphanous interacting protein), also known as WISH (WASP interacting SH3 protein) is a protein involved in cytoskeletal signaling, which regulates actin cytoskeleton and/or microtubule dynamics mainly through negative regulation of Rho [1]. More specifically, we previously reported that DIP/WISH functions as a negative regulator of Rho and a positive regulator of Rho GEF (guanine nucleotide exchange factor) [2]. We also recently showed that mouse embryonic fibroblast (MEF) cells from DIP/WISH-deficient (DIP/WISH-KO) mice have a narrow and long shape with many stress fibers under normal growth conditions [1]. In addition, the DIP/WISH-KO cells exhibited an activation of the Rho-ROCK pathway (particularly with regard to high ROCK activity), a decrease in *de novo *actin polymerization (especially polymerization requiring arp2/3 activity), and a decrease in cell motility and adhesion as evidenced by an *in vitro *analysis [1].

Spine-head volumes are known to change dynamically and reflect synapse strength. Moreover, spine-head volume changes accompany long-term functional plasticity of glutamatergic synaptic transmission, including long-term potentiation (LTP) and long-term depression (LTD) [3,4]. Actin reorganization represents a primary mechanism necessary to alter spine structures, and actin fibers are most concentrated in dendritic spines. Thus, actin is a critical regulator of spine and dendritic plasticity not only from a biochemical perspective but also from a physical point of view, both of which in turn could modulate LTP and LTD [5,6]. Involvement of the Rho family protein-mediated pathway in actin polymerization is supported by the fact that abnormalities in actin- and myosin-related proteins cause changes in spine morphology and mental retardation (MR) in mice and humans [7-9]. In particular, ROCK2 deficiency has been shown to cause MR [10]. Additionally, reports that SPIN90, the same protein as DIP/WISH, regulates dendritic spine morphology [11,12] and that breakpoint cluster region (BCR) and active BCR-related (ABR), both of which activate Rac, positively regulate LTP [13] support the importance of Rho family proteins for learning and memory via spine formation processes. Given these findings, other pathways altering Rho function could also affect learning and memory behaviors through changes in Rho and ROCK activities.

DIP/WISH is highly expressed throughout the brain including the hippocampus. To determine the involvement of DIP-dependent pathways in hippocampus-mediated events in mouse brain, we examined the expression of several synaptic proteins. In addition, we investigated the physiological function of hippocampal slices from DIP/WISH-KO mice and further conducted a series of behavioral studies with these mice to examine the physiological significance of DIP/WISH *in vivo*. Furthermore, actin is the main structural component of the stereocilia of cochlear (and vestibular) hair cells. The bundles of cross-linked actin provide the stiffness of the stereocilia which is important for the optimal function of this mechano-receptor. Acoustic signals act to deflect the stereocilia, and it is the relative motion between these stiff structures that opens mechanically gated ion channels to cause hair cell depolarization [14]. One can speculate that even very minor disruptions of the actin bundle integrity might cause a deterioration in threshold sensitivity of hair cells, and therefore auditory system function. Because of this we compared auditory function between the two genotypes using auditory brainstem evoked responses (ABRs).

## Results

### DIP expression in mouse hippocampus

**Figure 1:**
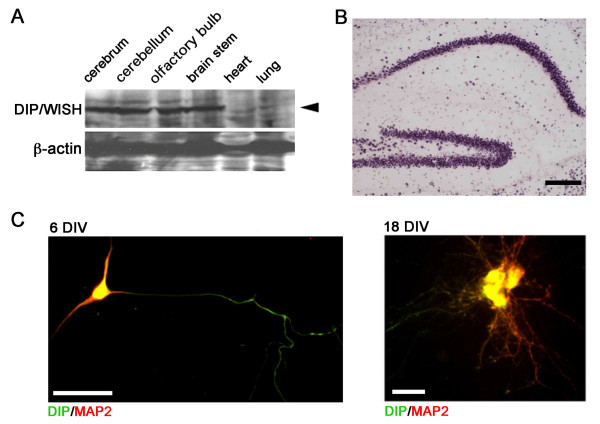
**DIP/WISH protein expression in mouse brain**. (A) Western blot analysis of DIP/WISH expression in several brain regions, heart, and lung of mice. (B) DIP/WISH mRNA expression in mouse hippocampus. (C) DIP/WISH protein expression in a dissociated mouse hippocampal neuron after 6 days in vitro (DIV, left) and 18 DIV (right). Scale bars indicate 500 μm (B), 50 μm (C, 6 DIV) and 10 μm (C, 18 DIV).

Although we previously reported that DIP/WISH is ubiquitously expressed, we found that DIP/WISH protein is expressed in various brain regions at a much higher level than observed in heart and lung (Figure 1A), suggesting an important role of DIP/WISH in the brain. The fact that spine remodeling, which involves actin reorganization, is critical for learning and memory led us to consider that DIP/WISH might be involved in hippocampal function. Therefore, we examined DIP/WISH expression in the hippocampus at both mRNA and protein levels. We observed dense DIP/WISH mRNA expression in the mouse hippocampal region (Figure 1B), while DIP/WISH protein expression with MAP2 was clearly observed not only in cell bodies, but also in dendrites and axons of dissociated hippocampal pyramidal neurons from new born mice (6 and 18 days after isolation) (Figure 1C). In particular, dense DIP/WISH expression in dendrites 18 days after isolation suggests an involvement of DIP/WISH in synaptic function.

**Figure 2:**
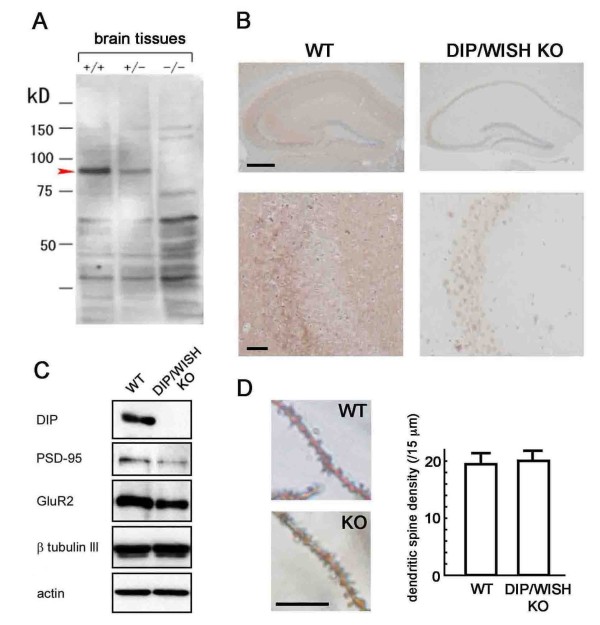
**Absence of DIP/WISH in DIP/WISH-deficient neurons**. (A) Absence of DIP/WISH protein in the brain from DIP/WISH-deficient mice. (B) Absence of DIP/WISH protein in the hippocampus from DIP/WISH-deficient mice. Brown (DAB) and blue (hematoxylin and eosin). (C) Comparison of the expression of several proteins in membrane fraction between WT and DIP/WISH-deficient hippocampal neurons. (D) Comparison of the number of spines in cultures of hippocampal neurons between WT and DIP/WISH-deficient mouse. Scale bars indicate 500 μm (B, upper), 50 μm (B, lower) and 5 μm (D).

Upon generating mice lacking the DIP/WISH gene, we first confirmed that DIP/WISH protein was not expressed in the brain of these mutant mice (Figure 2A). In contrast, DIP/WISH protein was broadly expressed in the wild-type (WT) mouse brain, especially in the CA3 region (Figure 2B). Strong DIP/WISH-like immunoreactivity was observed in the cell bodies of pyramidal neurons in WT hippocampus, and the protein was found accumulated in stratum lucidum (Figure 2B, left lower), consistent with data obtained from isolated pyramidal neurons (Figure 1B and 1C). Such strong DIP/WISH expression was absent in DIP/WISH-deficient hippocampal slices (Figure 2B, right). On the other hand, expression of other important proteins involved in synaptic function in hippocampus (including PSD-95 and GluR2) was not drastically changed in the hippocampus (membrane fraction) of DIP/WISH-deficient mice compared with WT controls although expression of PSD-95 and GluR2 looked a little reduced (Figure 2C). In addition, the number of spines calculated in Golgi-stained hippocampal samples did not differ between WT and DIP/WISH-deficient hippocampus (19.4 ± 0.9/15 μm and 20.0 ± 0.8/15 μm, respectively), although dendritic spine formation patterns looked a little different between the two genotypes (Figure 2D).

### Altered basal synaptic strength and presynaptic function in DIP/WISH-deficient mice

**Figure 3:**
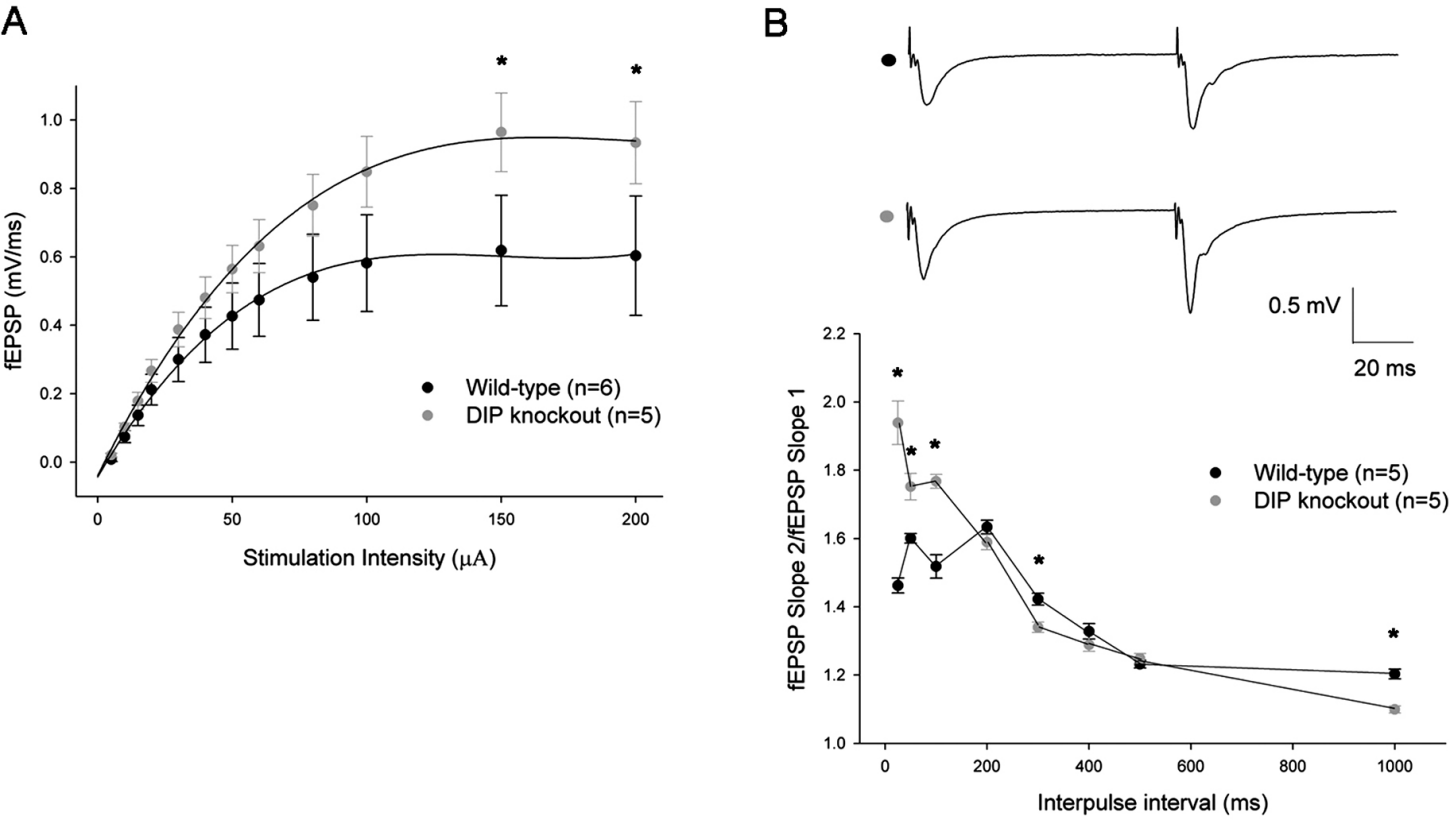
**Altered synaptic properties in DIP/WISH-deficientmice**. (A) Comparison of the field excitatory postsynaptic potential (fEPSP) slopes as a function of stimulation intensities revealed a significant difference (*P *< 0.01) between DIP/WISH knockout (n = 5) and wild-type slices (n = 6) in the range of higher stimulation intensities applied (150 and 200 μA respectively; as indicated by asterisks). (B) Paired-pulse facilitation (PPF), a short-term form of presynaptic plasticity, is notably altered in DIP knockout mice. PPF at intervals of 25, 50, 100, 300 and 1000 ms revealed a significant difference (*P *< 0.05; as indicated by asterisks) between DIP mutants (n = 5) and wild-type animals (n = 5).

To investigate the possibility of synaptic abnormalities as a result of DIP/WISH deficiency, we performed electrophysiological recordings in the CA1 region of the hippocampus. We first assessed basal synaptic strength by comparing changes in the size of recorded field excitatory postsynaptic potentials (fEPSPs) in response to a range of stimulation intensities. These studies revealed an increase in the size of fEPSPs in DIP/WISH-deficient mice (n = 5) in comparison to WT controls (n = 6), particularly over the higher stimulation intensities applied (Figure 3A; *P *< 0.01). This would indicate the importance of DIP/WISH in the physiological sustenance of basal synaptic transmission. Additionally, we also examined paired-pulse facilitation (PPF), a short-term form of neuronal plasticity that can be indicative of changes in pre-synaptic function (Figure 3B). These experiments revealed that PPF was significantly increased over shorter inter-pulse intervals (25, 50, 100, 300 ms; *P *< 0.05) in DIP/WISH mutants (n = 5) in comparison to WT slices (n = 5). This would suggest that pre-synaptic neurotransmitter regulation is significantly disrupted in DIP/WISH-deficient mice. Taken together, these results suggest the loss of DIP/WISH function has a considerable impact on basal synaptic physiology and function.

### Enhanced hippocampal long-term potentiation (LTP) in DIP/WISH-deficient mice

**Figure 4:**
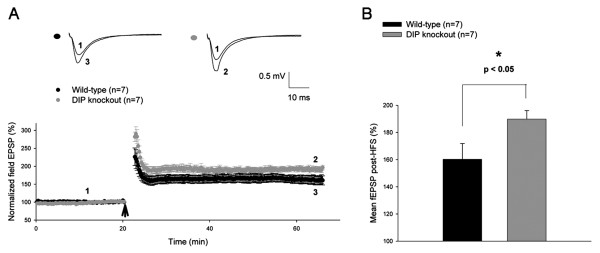
**Enhanced synaptic plasticity in DIP knockout mice**. (A) Long-term potentiation (LTP) induced by high-frequency stimulation (2 trains of 100 Hz with each train lasting 1 sec with an inter-train interval of 10 secs) was significantly enhanced (*P *< 0.05) in DIP knockout slices (190 ± 6.3%; n = 7) in comparison to wild-type slices (160 ± 11.6%; n = 7). (B) Comparison of the last 10 minutes of potentiation between DIP knockout mice and wild-type controls following the administration of HFS.

LTP in the hippocampal CA1 region is the most commonly studied form of synaptic plasticity, and is widely considered to be associated with learning and memory [15]. To test whether DIP/WISH plays a role in the regulation of this form of potentiation, we utilized a high-frequency stimulation (HFS) protocol of 2 trains of 100 Hz for the induction of plasticity in both WT and DIP/WISH-deficient animals. The administration of HFS resulted in an enhanced form of LTP in DIP/WISH mutants (Figure 4A), where the magnitude of potentiation was significantly elevated in comparison to WT controls (190 ± 6.3% for DIP/WISH-deficient mice; n = 7 versus 160 ± 11.6% for wild-type; n = 7; *P *< 0.05) (Figure 4B). These results clearly indicate the importance of DIP/WISH activity with regards to the maintenance of normal functional levels of synaptic plasticity.

### Performance in the Barnes circular maze test

**Figure 5:**
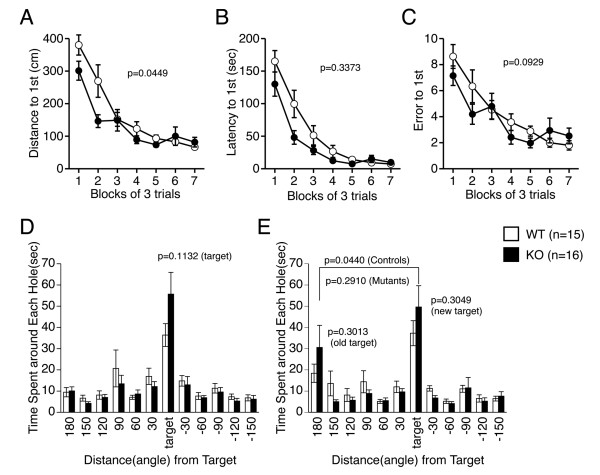
**Improved Barnes circular maze performance in DIP/WISH-deficient mice**. Measures of distance traveled (A), latency (B), and number of errors (C) related to the location of the escape tunnel during the course of the training period (days 1 - 7) in the Barnes circular maze test using 8 week-old wild-type (WT) and DIP/WISH KO (KO) mice. Distance was significantly shorter in KO mice. Time spent around each hole was measured in the probe trial (D). After reversal learning, probe test was conducted again (E), in which KO mice displayed behavioral perseveration.

Given the enhanced hippocampal LTP in DIP/WISH-deficient mice, we hypothesized that learning and memory may be altered in these mice. Therefore, to explore whether DIP/WISH is involved in long-term spatial memory, which is dependent on the functioning of the hippocampus, DIP/WISH-KO and WT mice (8 - 12 weeks old) were assessed on the Barnes circular maze [16]. DIP/WISH-KO mice showed no significant cognitive deficits in this test. All mice learned to locate the escape tunnel during the course of the training period (days 1 - 7), as indicated by a progressive reduction in escape latencies and number of errors. However, DIP/WISH-KO mice found the escape hole more rapidly over the course of the training period as indicated by a progressive reduction in distance traveled (F (1,29) = 4.393, genotype effect, *P *= 0.0449; genotype×trials interaction, *P *= 0.0274), latency (F (1,29) = 0.952, genotype effect, *P *= 0.3373; genotype×trials interaction, *P *= 0.1693) and numbers of errors to escape (F (1,29) = 3.020, genotype effect, *P *= 0.0929; genotype×trials interaction, *P *= 0.0115) although statistical significance was not achieved in the last two indices (Figure 5A-C). In the probe trial (transfer test) that was conducted 24 hours after the last training, there was not significant difference in time spent around the target hole (Figure 5D, P = 0.1132). In reversal learning, the target was moved 180° from the previous one, and the mice were given 12 training trials. Both genotypes learned the location of the new target to a similar extent (data not shown). One day after the last training of reversal learning, mice were again subjected to the probe test. Time spent around the new target and that around the previous one did not differ significantly between genotypes (Figure 5E, P = 0.3049, *P *= 0.3013, respectively). WT mice clearly showed a preference to the new target rather than the previous one (*P *= 0.0440). However, DIP/WISH-KO mice did not demonstrate a preference between the new target and the previous one (*P *= 0.2910), suggesting behavioral perseveration. We further performed the same behavioral experiments with an older group of mice (4 - 8 months old). However, DIP/WISH-KO mice in the older group did not show the difference (data not shown), suggesting that the DIP/WISH-deficient effects are more prominent during a restricted (younger) age period. No statistical differences were obtained in other series of behavioral experiments (data not shown).

### Auditory dysfunction in DIP/WISH-KO mice

**Figure 6:**
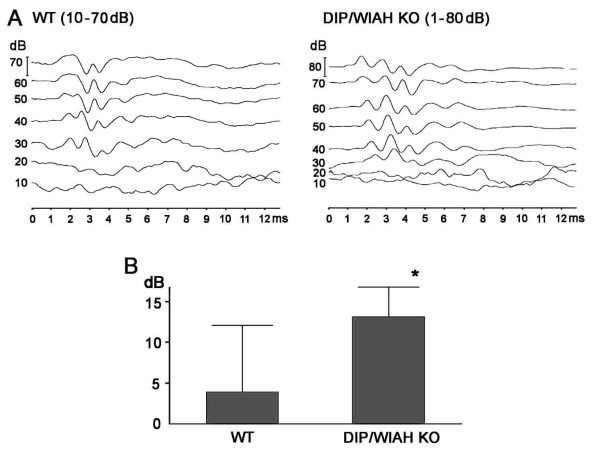
**ABR analysis of WT and DIP/WISH-deficient mice**. (A) ABR wave-forms were not substantially different between WT (10 to 80 dB) and DIP/WISH-deficient mice (0 to 80 dB) although there seemed to be a slight time elongation of wave-forms in DIP/WISH mice. (B) Average ABR thresholds for DIP/WISH-deficient mice were significantly higher (13.1 ± 1.3 dB) compared to those for WT mice (3.9 ± 2.7 dB, p < 0.05).

Auditory detection thresholds, as measured by ABRs to broad band click sounds, were significantly elevated in DIP/WISH-KO mice compared with WT mice (3.9 ± 2.7 dB for WT; n = 9, 13.1 ± 1.3 dB for DIP/WISH KO; n = 8, *P *< 0.05) (Figure 6). In an anatomical study using scanning electron microscopy (SEM) we systematically sampled basal, middle turn and apical areas of the sensory epithelium of the cochlea. However, we did not observe any obvious abnormality in hair cells or stereociliar morphology, and there was no hair cell loss (Additional file 1). This was perhaps not unexpected because whilst the auditory loss of 10 dB in the mutant mice was significant, it is a relatively mild deficit. The result implies that if there is any actin disorder in the stereocilia of hair cells, it is not substantial enough to be revealed by observations with SEM.

## Discussion

Our studies of field excitatory postsynaptic potentials and paired-pulse facilitation (Figure 3) suggest abnormalities of both pre- and post-synaptic functions in CA1 hippocampal neurons from DIP/WISH-KO mice. Consistent with these findings, an increased LTP was observed in hippocampal slice-recordings from DIP/WISH-KO mice compared with those from WT mice (Figure 4). The alteration in LTP observed in DIP/WISH-KO mice also raises the possibility of this factor being involved in the regulation of other prominent forms of synaptic plasticity. Towards this end, the opposing phenomenon of LTD [17] has been suggested to closely associate with LTP in regards to the maintenance of normal synaptic activity. The potentiation-enhancing effect of DIP/WISH seen in our study may imply that this protein could potentially reduce or block this type of depression. Further study of the possible role of DIP/WISH in LTD would be of significant interest. Given that both LTP and LTD can be induced through the actions of NMDA receptors, it would seem a natural extension of the present study to explore modifications at the level of the latter structures in respect to DIP/WISH deletion. This can be accomplished through the utilization of whole-cell recordings examining changes in NMDA-mediated currents in both wild-type and knockout slices, respectively. These electrophysiological changes obtained from the hippocampal neurons of the DIP/WISH-KO mice clearly match the behavioral phenotypes observed, in that DIP/WISH-KO mice found the escape hole in the Barnes maze more rapidly (Figure 5). Behavioral perseveration of the mutants in the probe test after reversal learning in the Barnes maze is also consistent with the results of electrophysiology. Taken together, we propose that DIP/WISH deletion improves the performance in the Barnes maze test through increased hippocampal long-term potentiation in mice.

Enhancement of the Rho-Rock pathway in the DIP/WISH-deficient cells [1] is most likely related to this phenomenon, and a decrease in *de novo *actin polymerization (especially polymerization depending on mDia- and WASP-mediated arp2/3 activity) could also be primarily involved. However, the significant differences revealed in the behavioral analysis were observed only at a certain age (up to 3 months of age), suggesting that DIP/WISH functions more critically in the relatively young mouse brain. The result that DIP/WISH-KO mice exhibited impaired auditory function compared with WT mice (Figure 6) without apparent morphological changes (Additional file 1) is in agreement with observation of deafness caused by a mutation of Dia in human [18] and with the enhancement of synaptic plasticity in DIP/WISH-deficient hippocampal slices.

Improvement of the performance in the Barnes maze test accompanied by increased Rho activity in DIP/WISH-KO mice is also consistent with the fact that both basal synaptic transmission and hippocampal long-term potentiation are impaired in ROCK2-deficient mice [8]. ROCK2-deficient neurons also show deficits in spine properties, synaptic density, and actin cytoskeletal development while these mutant mice are normal in gross brain anatomy [10] in a similar fashion to DIP/WISH-deficient mice [1]. Taking these results into account, it is likely that ROCK activity is elevated in DIP/WISH-deficient neurons. Recently, different MR-associated genes have been identified that encode modulators (GEF) of the Rho in mice and humans [13], which supports our result in which Rho activity is elevated. It has been established that loss or mutation of oligophrenin-1, the first identified Rho-linked MR protein [19], results in dendritic spine immaturity and altered pre-synaptic function causing a reduction of paired-pulse facilitation [20]. Oligophrenin-1 causes such effects through endocytosis of synaptic vesicles and post-synaptic AMPA receptor internalization [21,22]. Therefore, it is possible that DIP/WISH-deficiency produces the observed abnormality through the regulation of endocytosis and AMPA receptor internalization, and a more detailed analysis in this aspect will be important.

In our study, the levels of various synaptic proteins such as PSD-95 and GluR2 were not drastically altered in DIP/WISH-KO mice (Figure 2C). In addition, the number of spines calculated in Golgi-stained samples did not differ between WT and DIP/WISH-deficient hippocampus (Figure 2D). Since spatio-temporal changes in spine morphology in DIP/WISH-deficient hippocampi are highly expected from the observed effects in behavioral and electrophysiological analyses, conventional analysis with Golgi-staining may simple be insufficient to detect the changes. More detailed analyses with high-resolution imaging methods such as two-photon laser-scanning microscopy may be necessary to determine the effects of DIP/WISH on spine formation. Such an analysis would make the interaction of Rho-mediated actin organization and spine formation clearer.

## Conclusions

DIP/WISH deletion improves the performance in the Barnes maze test and increases hippocampal long-term potentiation in mice, an effect most likely due to synaptic changes associated with alterations in actin organization.

## Methods

### Animals

We generated DIP/WISH-KO mice and littermate WT control mice [1]. DIP/WISH-KO mice and WT control littermates were obtained by breeding heterozygote mice. Mice were group housed (2 - 4 mice per cage) in a room with a 12-hr light/dark cycle (lights on at 7:00 am) with access to food and water *ad libitum*. Behavioral testing was performed between 9:00 am and 6:00 pm. After the tests, the apparati were cleaned with diluted sodium hypochlorite solution to prevent a bias due to olfactory cues.

### Cell culture of dissociated hippocampal neurons

Mouse hippocampal neurons were prepared using a modified protocol from rat hippocampal culture methods [23]. In brief, hippocampi were dissected from P0 pups and dissociated using trypsin (0.25%) and trituration. Neurons were plated on poly-D-lysine-coated coverslips (15 mm round, Assistant, Sondheim, Germany) at a final density of 1-5 × 10^5 ^cells/coverslips in Neurobasal Medium (Invitrogen, Carlsbad, CA) with B27 supplement (Invitrogen, Carlsbad, CA). After 12 hours, coverslips were immersed in astrocyte-conditioned medium (Neurobasal Medium with B27 supplement). To prevent overgrowth of glia, neuron cultures were treated with cytosine arabinoside (5 mM; Calbiochem, La Jolla, CA) after incubation for 3 days *in vitro *(DIV).

### Biochemical analysis

Immunoblotting (IB) was performed using a membrane fraction of cells as previously mentioned [2]. For obtaining the membrane fraction, the cells were washed twice with ice-cold PBS containing 0.1 μM sodium orthovanadate (Na_3_VO_4_) and resuspended in TNE buffer (10 mM Tris-HCl, 150 mM NaCl, 1 mM EDTA, and complete EDTA-free protease inhibitor cocktail (PIC) (Roche), 1 μM Na_3_VO_4_). Samples were centrifuged for 15 min at 100,000 g. The pellets were then resuspended in TNE buffer with 1% Ipagel (NP-40) and sonicated for 30 s. Following centrifugation at 100,000 *g *for 30 min, protein concentrations of the supernatants were adjusted and they (200 μg) were subjected to IB. An anti-DIP/WISH antibody was made by us as shown previously [24]. First antibodies for β-tubulinIII, β-actin, PSD-95 and GluR2 are from Sigma (St. Louis), Sigma, Upstate (Lake Placid) and Santa Cruz Biotech. (Santa Cruz), respectively.

### In situ hybridization

Digoxigenin-labeled antisense/sense probes were used for *in situ *hybridization. Full coding region of mouse DIP cDNA was digested by *BamHI *and *XhoI*. The digested fragment was subcloned into pBluescriptSKII(+) *BamHI *and *XhoI *sites. After linearizing the plasmid (antisense: *BamHI*, sense: *XhoI*), digoxigenin-labeled antisense/sense probes were synthesized by RNA polymerase (antisense: T3 RNA polymerase, sense: T7 RNA polymerase). Detection of mRNA on cryo-sectioned tissues (14 mm) was performed by NBT/BCIP through alkaline phosphatase conjugated anti-DIG antibody (Roche).

### Immunohistochemistry

Immunohistochemistry was performed as previously described [25]. Briefly, adult mice (8 weeks old) were perfused with ice-cold 4% paraformaldehyde (PFA) into their left-ventricles. Brains were post-fixed with 4% PFA for 2 hours at 4°C, treated with 20% and 30% sucrose for several hours, embedded in OCT compounds (SAKURA, Tokyo, Japan), and then sectioned (10-14 μm thickness). The samples were pre-incubated with a blocking solution (3% bovine serum albumin and 0.3% Triton X-100 in phosphate-buffered saline (PBS)) for 1 hour, and then incubated overnight at 4°C in blocking solutions with rabbit polyclonal anti-DIP/WISH antibody (describe above). The signals were visualized with diaminobenzidine (DAB) staining by bright-field light microscopy.

For immunostainig of the dissociated hippocampal neurons, cells were incubated with anti-DIP/WISH and anti-MAP2 antibodies (Abcom, Cambridge, UK).

### Golgi staining

Golgi staining of hippocampal tissue from WT and DIP/WISH-KO mice was performed by using a section Golgi-impregnation procedure [26]. The number of spines was calculated in Golgi-stained hippocampal samples and expressed per 15 μm dendritic length.

### Behavioral analysis

We prepared a group consisted of an equivalent number of DIP/WISH-KO mice and WT control littermates (8 - 12 weeks old, WT, KO; n = 15, 16) for the behavioral tests. All the behavioral tests were conducted in a manner similar to those described previously [11]. All procedures involving the care and use of animals were carried out in accordance with institutional guidelines (National Institute for Physiological Sciences and the National Institute of Health guide for the care and use of laboratory animals and the Animal Care and Use Committee of Kyoto University Graduate School of Medicine). Raw data from the behavioral tests are shown in the mouse phenotype database (http://www.mouse-phenotype.org/).

The Barnes circular maze apparatus used was the one described by Takao et al. [16]. This task is similar to the Morris water maze, as both tests require an escape response. The Barnes maze test was chosen for this study since it does not involve swimming, which is a required component of the Morris water maze test. The maze used a dry, white circular platform that was raised 75 cm above the floor, was 1.0 m in diameter, and connected 12 holes equally spaced around the perimeter (O'Hara & Co., Tokyo, Japan). A black Plexiglas escape box (17 × 13 × 7 cm), which had paper cage bedding on its bottom, was located under one of the holes. The hole above the escape box represented the target, similar to the hidden platform in the Morris task. The location of the target was consistent for a given mouse, but was randomized across mice. The maze was rotated daily, with the spatial location of the target unchanged with respect to the visual room cues, to prevent a bias based on olfactory or proximal cues within the maze. The first training of the first group was started when WT mice and DIP/WISH-KO mice were 18 - 23 weeks old. In order to familiarize mice with the maze and the existence of the escape box, they were subjected to two habituation sessions prior to the beginning of testing. Three trials per day were conducted for 7 successive days in the beginning. Distance, latency, and numbers of errors to reach the target hole were recorded. Genotype effects on these indices were analyzed by two-way repeated ANOVA. One day after the last training, a probe test was conducted without the escape box, to confirm that this spatial task was acquired based on navigation using distal environmental room cues. Mice were allowed to freely explore the maze for 3 min. Time spent around each hole was recorded. A single training trial was conducted immediately after the probe test. An additional probe test (retention test) was conducted one week after the last training trial. Latency to reach the target hole and time spent around each hole were recorded by video tracking software (Image BM; see ref. [16]). One week after the memory retention test, the escape box was moved to a new position opposite to the original (reversal learning). Mice were then tested on five successive days to locate the new position of the escape hole using the same procedure as described above.

### Preparation of hippocampal slices

The preparation of brain slices has been previously described [27]. DIP-deficient and wild-type littermate mice (8-weeks old, backcrossed more than 7 times) were sacrificed using cervical dislocation, after which the brain was extracted quickly from the skull utilizing a curved spatula. The extracted brain was then transferred to ice-cold ACSF containing (in mM) 120 NaCl, 2.5 KCl, 1.3 MgSO_4_, 1.0 NaH_2_PO_4_, 26 NaHCO_3_, 2.5 CaCl_2_, and 11 D-glucose (where it was kept for a period of 1-2 minutes). Following this, the brain was then moved to a vibratome (752 M Vibroslice, Campden Instruments, Lafayette, IN, U.S.A.) where hippocampal slices (400 μm) were sectioned and collected in the presence of ice-cold ACSF. The slices were then allowed to recover in a submerged holding chamber bubbled with carbogen (95% O_2_/5% CO_2_). The time of recovery was at least 1 hour before the initiation of recording experiments.

### Extracellular fEPSP electrophysiological recordings

After a recovery period of at least 1 hour in the submerged holding chamber, a single slice was subsequently transferred to the recording chamber where it underwent submersion and superfusion with 95% O_2_-5% CO_2 _saturated artificial cerebral spinal fluid (ACSF, 2 ml/min) at a controlled temperature of 28°C. The ACSF contained (in mM) 120 NaCl, 2.5 KCl, 1.3 MgSO_4_, 1.0 NaH_2_PO_4_, 26 NaHCO_3_, 2.5 CaCl_2_, and 11 D-glucose. For recording of field EPSPs, the glass recording pipette (3 MΩ) was filled with ACSF solution.

Synaptic responses were evoked by bipolar tungsten electrodes located 200-400 μm from the cell body layer of the hippocampal CA1 region. fEPSPs were measured by utilizing the rising phase slope between 5% and 60% of the peak response. All data acquisition and analysis were achieved through use of the pCLAMP 7 software (Axon instruments). Paired-pulse facilitation (PPF) was recorded with the first and second responses being separated by a range of stimulation intervals. The ratio between the magnitudes of the slope of the succeeding response to the slope of the initial response was then calculated. For LTP studies, a high-frequency stimulation (HFS) induction protocol using 2 trains of 100 Hz at 10 second intervals (with each train lasting 1s) was utilized following a stable baseline period. n corresponds to the specific number of hippocampal slices used in each experimental group. Customarily, one slice was used per mouse during experiments. For representation of average data, data underwent normalization to the average of the baseline responses unless otherwise stated. The shown representative traces of fEPSPs were averages of five successive sweeps during experimental recordings. Representative traces for PPF studies were obtained at the 100 ms interval. For assessment of the LTP size, the means of potentiation during the last 5-10 minutes of field recordings in both wild-type and mutant groups were compared statistically by the use of Student's *t*-test. For analysis of basal synaptic strength, experimental results were examined by comparing the recorded means at the indicated stimulation intensities between wild-type and mutant groups through Student's *t*-test. For PPF studies, data obtained at the stated interpulse interval recordings were weighed between wild-type and mutants via Student's *t*-test. In all the above cases, a P-value was obtained where *P *< 0.05 was considered significant. All experimental protocols were approved by The Hospital for Sick Children Animal Care Committee.

### Test of auditory function

We used ABR testing to evaluate auditory thresholds. DIP/WISH-KO and WT mice at 12 weeks of age were anesthetized with an i.p. injection of pentobarbital sodium (Nembutal), and anesthesia was supplemented during the course of measurement as necessary. The electrodes were subcutaneously inserted at the vertex (positive), left or right ear (mastoid; negative) and opposite mastoid (ground). In a calibrated, closed sound system, broadband click stimuli were presented at levels from 0 - 80 dB sound pressure level (SPL). Evoked responses were amplified (x10k), filtered with a band pass of 200 Hz to 3 kHz and averaged with 500 sweeps. Typical ABR waveforms evoked at increasing stimulus levels are shown in Figure 6. Experimental protocols were approved by The Hospital for Sick Children Animal Care Committee.

### Scanning electron microscopy

Inner eras were fixed in 2.5% glutaraldehyde in PBS for at least 1 hour. The sensory areas were microdissected, dehydrated in graded ethanol solutions and critical point-dried from liquid CO2. Procedures for scanning electron microscopy were as described [28].

## Competing interests

The authors declare that they have no competing interests.

## Authors' contributions

SA carried out electrophysiological experiments and drafted the manuscript. KK carried out biochemical experiments. KK and KT carried out behavioral studies and analysis. KT drafted the manuscript. JN and RVH carried out auditory function studies and scanning EM studies. MM carried out a Golgi-staining study. KS carried out an in situ-hybridization study and immunostaining. TM organized behavioral studies and analysis. ZJ participated the study design and conducted the electrophysiological studies. MWS participate in the study coordination and discussion. MT drafted the manuscript. TF-T conceived the study, participated in its design, carried out biochemical experiments, and drafted the manuscript. All authors read and approved the final manuscript.

## Supplementary Material

Additional file 1 **Histological comparison of the cochlea from WT and DIP/WISH-deficient mice using scanning electron microscopy**. There were no obvious differences in cochlear structure and hair cell morphology between WT and DIP/WISH KO. Scale bars indicate 100 μm (apex), 20 μm (mid-turn) and 50 μm (base). Click here for file
