# Supplementary figures and images for: DIP/WISH deficiency enhances synaptic function and performance in the Barnes maze
Source: Mol Brain. 2011 Oct 21;4:39. doi: 10.1186/1756-6606-4-39 (PMC3208581; doi:10.1186/1756-6606-4-39)

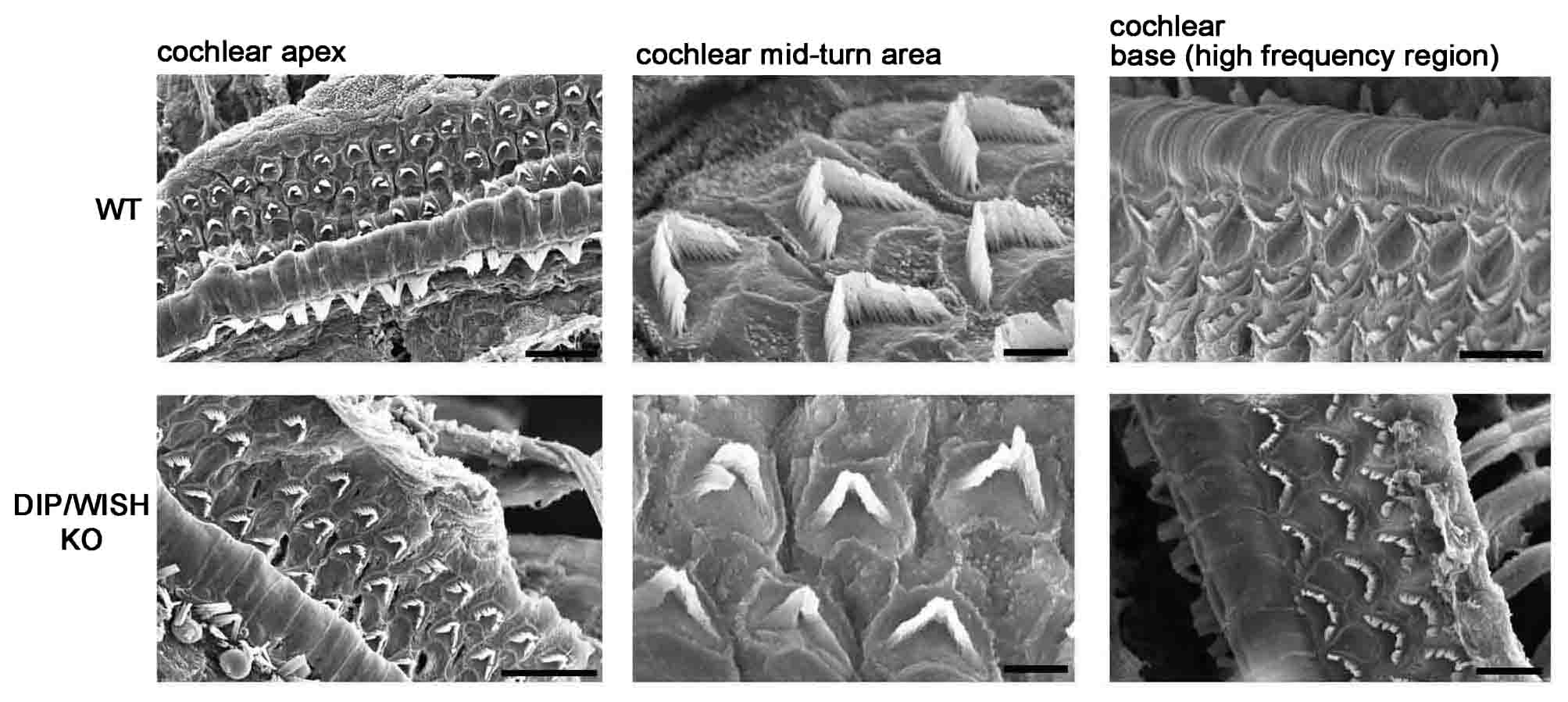

Supplement: Additional file 1 — Histological comparison of the cochlea from WT and DIP/WISH-deficient mice using scanning electron microscopy. There were no obvious differences in cochlear structure and hair cell morphology between WT and DIP/WISH KO. Scale bars indicate 100 μm (apex), 20 μm (mid-turn) and 50 μm (base). [file 1756-6606-4-39-S1.JPEG]
